# Supplementary material for: Orthogonal analysis of mitochondrial function in Parkinson’s disease patients
Source: Cell Death Dis. 2024 Apr 3;15(4):243. doi: 10.1038/s41419-024-06617-6 (PMC10991487; doi:10.1038/s41419-024-06617-6)
Supplement: Supplementary file 1 — Supplemental material [file 41419_2024_6617_MOESM1_ESM.docx]

Supplementary material legends

**Supplementary table 1:**

Demographic table summarizing number, age and sex of PD patients and healthy controls groups.

**Supplementary table 2:**

Table of clinical severity. Patients are divided in four groups of increasing and comparable disease severity based on motor (UPDRS-III) and non-dopaminergic (SENS-PD) clinical scales, and upregulation of mitochondrial respiration in galactose conditions.

**Supplementary table 3:**

PD patients’ erythroblasts maximal mitochondrial respiration comparisons between clinical severity groups. The differences in maximal respiration in the groups with the mildest and most severe presentation (i.e. severity 1 vs. severity 4) and between the severity groups 3 and 4 were close to statistical significance (p=0.05281 and p=0.05429 respectively).
